# The Effects of Dienogest on Macrophage and Natural Killer Cells in Adenomyosis: A Randomized Controlled Study

**DOI:** 10.22074/ijfs.2018.5137

**Published:** 2017-10-14

**Authors:** Saowapak Prathoomthong, Yada Tingthanatikul, Srithean Lertvikool, Nittaya Rodratn, Wanwisa Waiyaput, Kanthanadon Dittharot, Morakot Sroyraya, Areepan Sophonsritsuk

**Affiliations:** 1 Department of Obstetrics and Gynecology, Faculty of Medicine, Ramathibodi Hospital, Mahidol University, Bangkok, Thailand; 2 Reproductive Endocrinology and Infertility Unit, Department of Obstetrics and Gynecology, Faculty of Medicine, Ramathibodi Hospital, Mahidol University, Bangkok, Thailand; 3 Office of Research Academic and Innovation, Faculty of Medicine, Ramathibodi Hospital, Mahidol University, Bangkok, Thailand; 4 Department of Anatomy, Faculty of Science, Mahidol University, Bangkok, Thailand; 5 Mahidol University Nakhon Sawan Campus, Nakhon Sawan, Thailand

**Keywords:** Adenomyosis, Dienogest, Macrophages, NK Cells, Progestins

## Abstract

**Background:**

Progestin has been used for symptomatic treatment of adenomyosis, although its effect on the immune system has not been studied. The aim of this study was to investigate the changes of macrophage and natural killer (NK) cell infiltration in tissues obtained from women with adenomyosis who did or did not receive oral progestin dienogest.

**Materials and Methods:**

In this randomized controlled clinical trial study, 24 patients with adenomyosis who re- quired hysterectomy were enrolled. Twelve patients received dienogest 28-35 days before surgery, and the other 12 patients were not treated with any hormones. The endometrial and myometrial tissue samples were immediately collected after hysterectomy, and immunohistochemistry for a macrophage marker (CD68) and a NK cells marker (CD57) was performed.

**Results:**

The number of CD57 cells was significantly increased in endometrial glands of the treated group compared to the untreated group (P=0.005) but not in stroma in the endometrium of the treated patients (P=0.416). The differ- ence in the number of CD68 cells was not statistically significant between treated and untreated groups in the endo- metrial glands (P=0.055) or stromal tissues (P=0.506).

**Conclusion:**

Administration of oral progestin dienogest to patients with adenomyosis increased the number of uterine infiltrating NK cells in glandular structure of eutopic endometrium. The differential effects of progestin on NK cells depended on the site of immune cell infiltration. The effects of oral progestin on uterine NK cells in adenomyosis have the potentials to be beneficial to pregnancies occurring following discontinuation of treatment in terms of embryo im- plantation and fetal protection (Registration number: TCTR20150921001).

## Introduction

Adenomyosis is a common gynecologic disease in premenopausal and late reproductive age women. Frequent symptoms include progressive dysmenorrhea, chronic pelvic pain, dyspareunia, abnormally heavy menstrual bleeding, and interval menstrual bleeding. The severity and frequency of the symptoms correlate with the extent and depth of the ectopic endometrium in the myometrium, which follows the endometriosis pathology and progression of the disease (1). Generally, diffuse globular enlargement is the common sign of adenomyosis and the size of uterus is typically not larger than 12 weeks gravid size. This enlargement of the uterus is due to invasion of the myometrium by endometrial glands only be made at histopathology following hysterectomy. 

Currently, the etiology and pathology of adenomyosis have yet to be elucidated, however, many theories have been proposed; for example, the invagination of basalis endometrium into the myometrium and the subsequent inflammation. Adenomyosis uteri repetitively exhibit exaggerated and non-synchronized uterine contractions, which induce a micro-fracture at the endometrial-myometrial junction zone (EMJZ). This micro-trauma at EMJZ causes the displacement of endometrium into the surrounding myometrium, where the myometrial cells proliferate and undergo metaplasia, resulting in the thickening of EMJZ (3, 4). The aberrant immunologic activity in adenomyosis is seen by immunohistochemistry with an increase in the number of infiltrating macrophages. These could activate T and B cells to produce antibodies and cytokines that may destroy the EMJZ (5). Macrophages transform from the circulatory monocyte cells and serve many functions including phagocytosis, antibodydependent cell-mediated cytotoxicity, and presenting antigens to lymphocytes. Natural killer (NK) cells, on the other hand, are cytotoxic lymphocytes that play a role in innate immune response. They can remove infected cells without recognition, and thus the killing process is an immediate response to virus-infected cells and tumor formation (6, 7).

Abnormal immune responses appear to play a role in adenomyosis. Expression of human leukocyte antigen (HLA) class II is recognized as the first step in the activation of macrophages and/or T lymphocytes by presenting foreign antigens to these cells. HLA class II expression is shown to be increased in the glandular cells of eutopic and ectopic endometrium of adenomyosis (6, 7). At this point, the definitive treatment for adenomyosis is hysterectomy (5), but it is not the treatment of choice for patients who wish to remain fertile. Oral contraceptive pills, high doses of progestin, levonorgestrel-releasing intrauterine device (LNGIUD), gonadotropin releasing hormone agonist (GnRH-a), and danazol are hormonal treatment options for both adenomyosis and endometriosis (1). The use of pre-operative GnRH agonists in patients with myomas, endometriosis and adenomyosis was tested by Khan et al. (8). They found a decrease in MCP-1 level, as well as a reduction in the number of macrophages in endometrial and myometrial layers of patients with adenomyosis compared to an untreated group. Progestin is considered a good choice for long-term treatment of adenomyosis because it causes minimal side effects. The administration of a progestin, depending on the dose, may not cause a hypoestrogenic state.

Four generations of synthetic progestogens are now available to clinicians. A new progestin called dienogest (DNG) ( 17a-cyanomethyl-17b-hydroxyestra-4, 9-dien bioavailability and strong progestational effect due to its high selectivity to the progesterone receptor. It has been reported as one of the progestogens employing the strongest action on the endometrium (10). Its half-life is 9-10 hours, and it is approximately 90% secreted by the kidneys. The therapeutic dose of DNG for treatment of endometriosis is 2 mg daily providing a blood level of 10-7 mol/L. Although the blood level of dienogest after oral administration is very low, the symptoms of endometriosis can be cured (9). Dienogest has been shown to slightly increase the number and macrophages (11). The objective of our study was to investigate the effect of DNG on immune cells focusing on macrophages and NK cells in women with adenomyosis. 

## Materials and Methods

A randomized control trial study was conducted from 1 February 2015 to 31 May 2016 at the Department of Obstetrics and Gynecology, Faculty of Medicine, Ramathibodi Hospital, Mahidol University, Bangkok, Thailand. The study was approved by the Ethical clearance Committee on human rights related to researches involving human subjects ( pro women aged 20-50 years, suffering from dysmenorrhea with or without hypermenorrhea, undergoing either abdominal hysterectomy or laparoscopic hysterectomy for adenomyosis, and diagnosed adenomyosis by ultrasonography. The exclusion criteria were patients who had used i. Pill or oral exogenous hormones for 3 months or ii. GnRH agonist or depot medroxyprogesterone acetate injection for 6 months before surgery. Twenty-four eligible patients were recruited into the study. The ultrasonographic diagnostic criteria for adenomyosis included a globular shape with diffuse uterine enlargement, myometrial anterior-posterior asymmetry, poorly described areas in the myometrium, a heterogeneous myometrial echotexture, sub-endometrial echogenic linear striations with or without small myometrial cysts, or hemorrhagic foci within the heterotopic endometrial layer (12). The sample size was calculated according to measurement outcomes and the mean number of NK cells and macrophages from Khan’s study with ple size was 12 for each arm. Therefore, a total of 24 subjects were needed for this present study. 

The subjects were divided into two groups via block randomization with a block size of 4. A group of 12 patients received 2 mg/day dienogest for 28-35 days before surgery, and the other 12 underwent operation without prescribing the drug (control group). The phase of the menstrual cycle was determined by the subjects’ last menstrual period and confirmed by endometrial histology. Patients with liver disease, kidney disease, autoimmune disease, coagulopathy, endometriotic cysts, or under hormonal therapy were excluded. Exclusion of patients with pelvic endometriosis was not possible; therefore, it was the potential limitation for the present study.

### Tissue collection and preparation

The myometrial tissue had gross features of adenomyosis, that is, hypertrophic swirls of smooth muscle separating duller prominent trabeculated patterns and gray foci of endometrium. The tissue was excised in 1cmx1cmx1cm dimentions immediately after hysterectomy. The endometrial tissue sample was also collected in block pattern size 1 cm×1 cm×1 cm from the myometrial layer. All collected sections of the samples were then prepared for subsequent histopathological and immunohistochemical studies. 

### Immunohistochemistry

Immunohistochemical analysis was performed with anti- CD68 antibody for macrophages and anti-CD57 and anti- CD56 for NK cells. Both primary rabbit monoclonal antibodies clone PGM-1 and clone 123C3.D5 against CD68 and CD57, respectively, were from DAKO (Glostrup, Denmark), and the primary mouse monoclonal antibody clone NK-1 against CD56 was from Thermo Fisher Scientific (Waltham, USA). Primary antibodies CD68, CD57, and CD56 were used at a dilutions of 1:100, 1:150, and 1:2400, respectively. The 3-μm thick parafﬁn-embedded tissue sections were deparafﬁnized in xylene and then rehydrated. Slides were incubated for 60 minutes at 60°C and treated with Bond Dewax Solution (Leica Biosystems, Bannockburn, IL). Epitope retrieval was performed by incubating the slides in Bond Epitope Retrieval Solution for 20 minutes at 100°C. Immunohistochemical analysis was performed using the Bond Polymer Refine Detection kit (Leica Biosystems, Bannockburn, IL), a 3-step indirect immunoperoxidase technique. Briefly, primary antibody was applied for 45 minutes at room temperature. Peroxide block (3% hydrogen peroxide) was then applied for 5 minutes and rinsed with Bond Wash Solution. Post Primary Polymer was applied for 9 minutes. Polymer Poly- HRP IgG was applied for 7 minutes and rinsed with Bond Wash Solution and deionized water, then diaminobenzidine chromogen was applied for 4 minutes. Slides were counterstained with hematoxylin for 5 minutes. Appendix tissue was used as positive control. The negative control showed an absence of specific staining (12).

The number of CD68 and CD57 brown spots were counted in 20 different fields (200×200 microns) for each person (magniﬁcation: ×200) under light microscopy. The number of CD68 and CD57 positive cells were calculated and expressed as the mean positive cells per mm ^2.Theresultsineachbiopsy^er who did not know the patient’s history. Double-labeling immunohistochemical method for CD57 and CD56 was also performed. The primary antibody anti-CD57 was the first antibody identified by diaminobenzidine chromogen, while the primary antibody anti-CD56 was the second antibody identified by mixed red refine chromogen. The procedure was similar to the described protocol above including deparaffinization, epitope retrieval and a 3-step indirect immunoperoxidase technique. However, before counterstaining each slide with hematoxylin, the second antibody (anti-CD56 primary antibody) was applied for 40 minutes at room temperature. Post Primary Polymer AP was applied for 20 minutes. Polymer Poly-AP IgG was applied for 20 minutes and rinsed with Bond Wash Solution and deionized water before the Mixed were then counterstained with hematoxylin for 5 minutes. 

### Statistical analysis

Each parameter is presented as either the mean ± SD or medians (25, 75%) depending on the distribution of data. The clinical characteristics were compared by chi-square test or student’s t test for differences between two groups. The differences in numbers of macrophages and NK cells between the two groups were analyzed by the non-parametric Mann-Whitney U-test; a value of P<0.05 was considered statistically significant. The data were analyzed by IBM SPSS Statistics for Windows, version 19.0 (Armonk, NY: IBM Corp).

## Results

**Fig.1:**
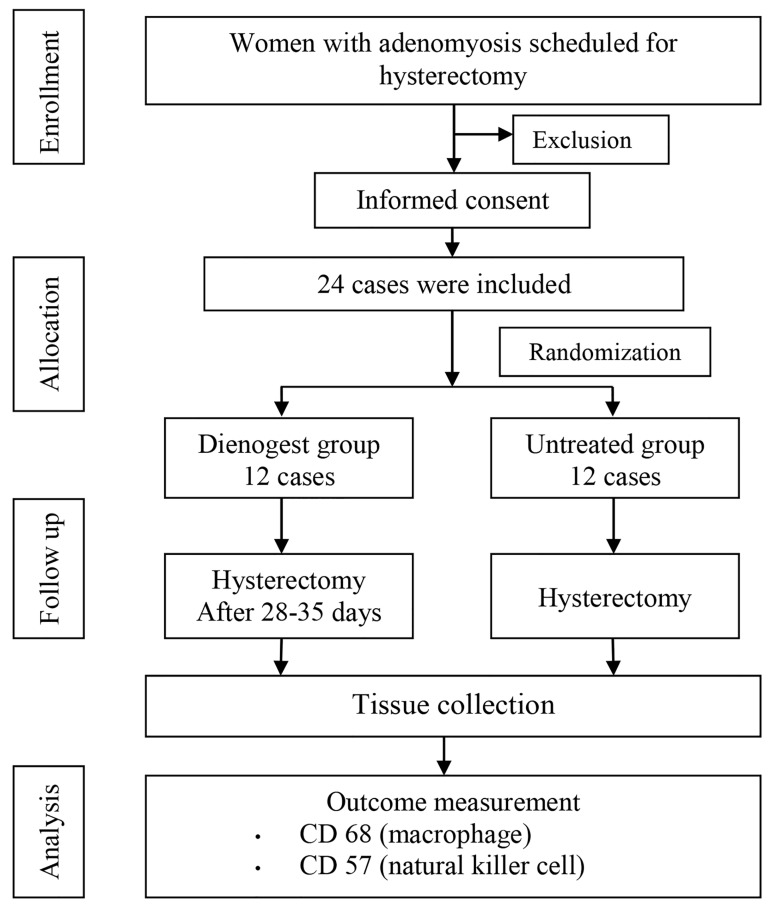
Study design for the effects of dienogest on macrophages and natural killer cells in adenomyosis clinical trials.

Twenty-four elibible patients were enrolled and randomly divided into treated and untreated groups. There were no dropouts after treatment of dienogest for 28-35 days, therefore, complete data from 24 participants were available for analysis (Fig .1). The demographic characteristics of the participants; for example, age, BMI, indication for and type of surgery were not significantly different (Table 1). The number of participants with proliferative and secretory menstrual phases in the DNG and untreated groups were not statistically different.

**Table 1:** Demographic data of women with adenomyosis


Characteristic	Dienogest n=12 mean ± SD	Untreated n=12 mean ± SD	P value

Age (Y)	43.8 ± 5.16	45.4 ± 4.48	0.458
BMI (kg/m^2^)	25.9 ± 4.78	24.6 ± 2.99	0.878
Phase	n (%)	n (%)	
Proliferative phase	5 (41.66%)	6 (50%)	1.000
Secretory phase	7 (58.33%)	6 (50%)	1.000
Indication for surgery			
Dysmenorrhea	7 (58.33%)	8 (66.66%)	1.000
AUB	5 (41.66%)	4 (33.33%)	1.000
NSAIDs usage	11 (91.66%)	10 (83.33%)	1.000
Type of operation			
Laparotomy	11 (91.7%)	12 (100%)	1.000
Laparoscopy	1 (8.3%)	0	1.000


BMI; Body mass index, AUB; Abnormal uterine bleeding, and NSAIDs; Nonsteroidal antiinflammatory drug.

### Effect of dienogest on endometrial gland and stroma of endometrium

**Fig.2:**
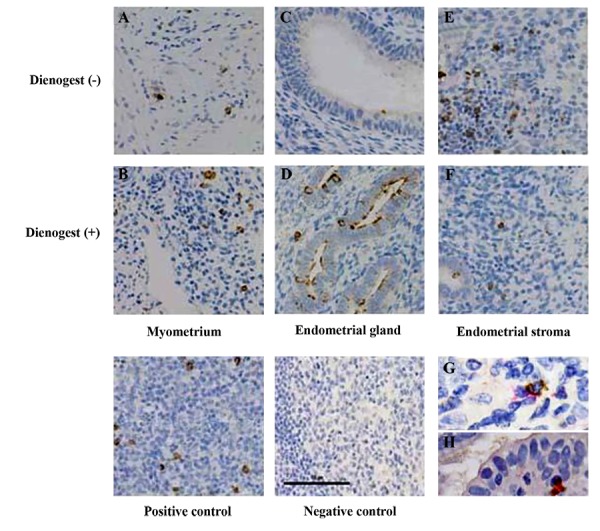
Immunohistochemistry staining for CD57 in eutopic and ectopic endometrium. The accumulation of CD57 is indicated by a dark brown coloration in the nucleus; all nuclei were counter-stained with hematoxylin. **A.** Ectopic endometrium in myometrium of adenomyosis women in untreated group, **B.** Ectopic endometrium in myometrium of adenomyosis women in dienogest-treated group, **C.** Endometrial gland in endometrium of untreated group, **D.** Endometrial gland in endometrium of dienogest-treated group, **E.** Endometrial stroma in endometrium of untreated group, **F.** Endometrial stroma in endometrium of dienogest-treated group, **G.** Double staining for CD57 and CD56 in endometrium, and **H.** Double staining for CD57 and CD56 in myometrium.

**Fig.3:**
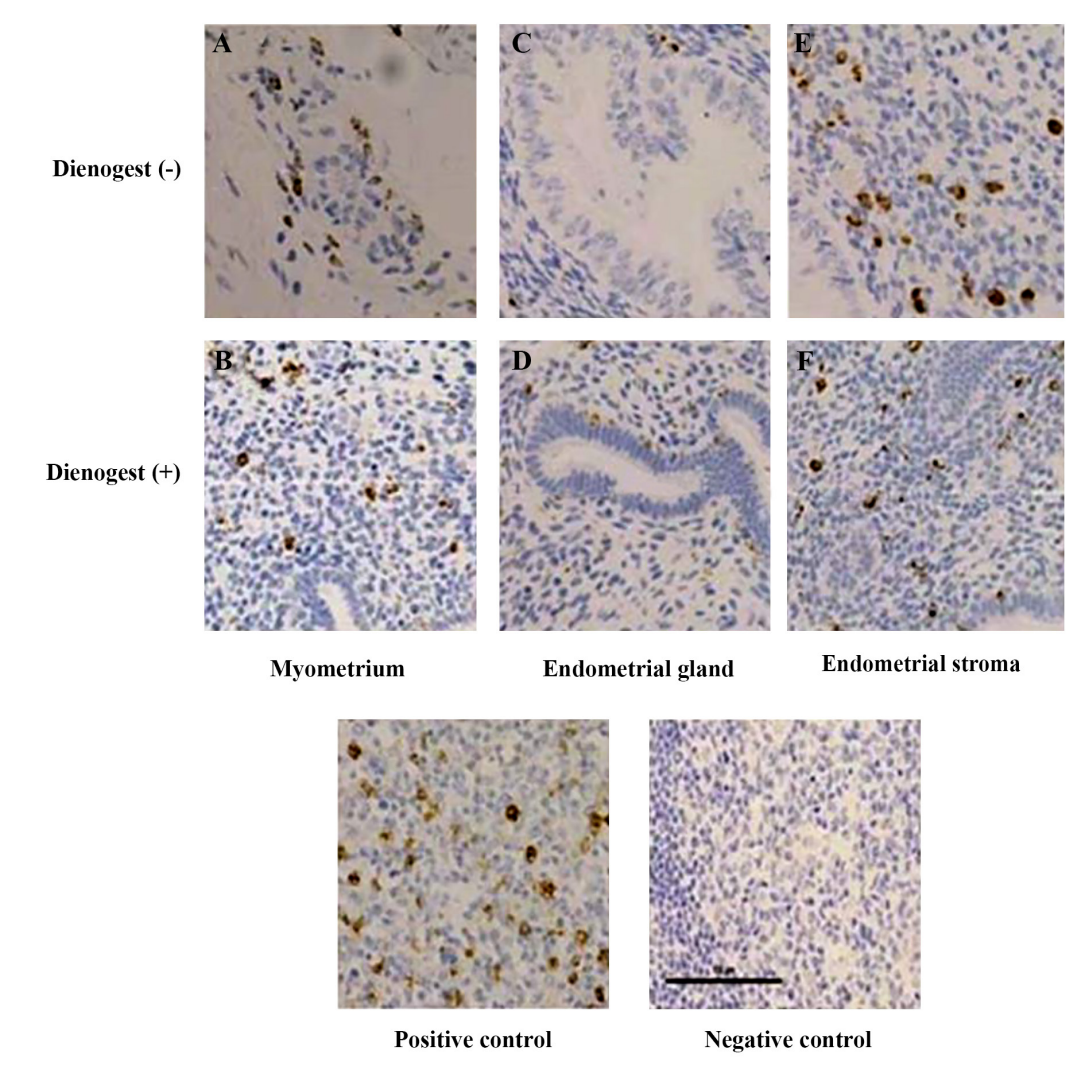
Immunohistochemistry staining for CD68 in eutopic and ectopic endometrium. The accumulation of CD68 is indicated by a dark brown coloration in the nucleus and all nuclei were counter-stained with hematoxylin. A. Ectopic endometrium in myometrium of adenomyosis women in untreated group, B. Ectopic endometrium in myometrium of adenomyosis women in dienogest-treated group, C. Endometrial gland in endometrium of untreated group, D. Endometrial gland in endometrium of dienogest-treated group, E. Endometrial stroma in endometrium of untreated group, and F. Endometrial stroma in endometrium of in denogest-treated group.

NK cell infiltration, as shown by CD57-positive brown spots (Fig .2), was significantly increased in glands in treated versus untreated groups [P=0.005, median (range 25, 75%), 4.37 (0.31, 14.68) vs. 0 (0, 0)] (Fig .2D), but not in stroma of the endometrium of the treated group [P=0.416, median (range 25, 75%), 21.87 (10, 68.81), 10 (8.75, 15.93)] (Fig .2F, Table 2). NK cells were confirmed by double immunostaining for CD57 and CD56 (Fig.2G, H). Macrophage infiltration, as demonstrated by CD68- positive brown spots (Fig .3), was also increased, but was not statistically significant in treated versus untreated groups [P=0.055, median (range 25, 75%), 4.37 (0, 9.06), 0 (0, 1.56)] (Fig .3D, Table 2).

**Table 2:** Macrophage and natural killer (NK) cell infiltration in endometrial glands and stroma in endometrial tissue in adenomyosis patients


	Glands			Stroma		
	Untreated n=12	Dienogestn=12	P value	Untreated n=12	Dienogest n=12	P value

Macrophage (cells/mm^2^)	0(0-1.56)	4.37(0-9.06)	0.055	44.37(22.18-50.93)	64.37 (20.62-92.5)	0.506
NK cells (cells/mm^2^)	0(0)	4.37(0.31-14.68)	0.005	10(8.75-15.93)	21.87 (10-68.81)	0.416


Data as presented as [median (range 25-75%)].

### Effect of dienogest on the myometrium surrounding the ectopic endometrium

Infiltration of macrophages and NK cells in the myometrium of women with adenomyosis was also investigated. No differences were seen between DNG-treated and untreated groups (Figes.2, 3, Table 3).

### The decrease of endometrial thickness after dienogest treatment

The uterine endometrial thickness was more significantly reduced after DNG treatment for 30 ± 2.76 days (mean ± SD) when compared to the untreated group [median (25, 75%), 37.5 (33.37, 60.12), 94.5 (67.75, 143.25), respectively] (P=0.021).

**Table 3:** Macrophage and natural killer (NK) cells infiltration in ectopic endometrium in myometrial tissue in adenomyosis patients


	Untreatedn=12	Dienogestn=12	P value

Macrophages (cells/mm^2^)	8.75 (4.06-17.18)	5 (3.75-13.43)	0.663
NK cells (cells/mm^2^)	5.62 (4.68-19.06)	11.25 (9.68-16.87)	0.354


Data as presented as [median (range 25-75%)].

### Discussion

This randomized controlled trial compared the numbers of infiltrating macrophages and NK cells in the eutopic and ectopic endometrium in women with adenomyosis after either one month of treatment with DNG or no treatment. The results showed that oral DNG increased the number of NK cell infiltration in glandular structure of eutopic endometrium but not in ectopic endometrium. However, no differences were observed in the number of macrophages and NK cells in stromal tissue of eutopic endometrium or ectopic endometrial tissue of the two study groups. This suggests that progestin has a direct effect on endometrium in addition to its systemic effect, thus may improve local immunity in the endometrium.

The histopathological characterization of adenomyosis is similar to endometriosis, except for the site of endometriotic lesions. Immune dysfunction and inflammation play significant roles in both endometriosis and adenomyosis. Various immune alterations occur in endometriosis that are also present in adenomyosis. We noted an increase in the number and activity of macrophages secreting proinflammatory cytokines interleukin-1 (IL-1) and tumor necrosis factor (TNF) in women with endometriosis. It has been shown that patients with adenomyosis have both HLA-DR and HLA-G expression in eutopic and ectopic endometrium (7, 14). The expression of HLA-G corresponding to major histocompatibility complex (MHC) class I increases the survival of endometrial cells from the host’s immunosurveillance (13). Ota and Igarashi (7) and Ota et al. (15) reported an increased number of macrophages in ectopic and eutopic endometrium in women with adenomyosis. Recently, Zhihong et al. (16) did a prospective case-control study by collecting the endometrial tissue from patients with adenomyosis during the implantation window after ovarian stimulation, and then performed immunohistochemistry and real time-polymerase chain reaction (RT-PCR). The results showed an increase in cytokine IL-6, monocyte chemoattractant protein-1 (MCP-1) and the number of macrophages. MCP-1, which is produced by uterine NK cells is a cytokine that induces the migration of immune cells to target tissue.

NK cells play a role in destroying viruses and tumor cells but not normal host cells. The killing activity depends on the activity of killer cell inhibitory receptors (KIRs) and MHC molecules (17). The reduction of NK cell activity in peripheral blood and peritoneal fluid has been shown in endometriosis (18, 19). The NK cell cytotoxicity may also be reduced in eutopic endometrium in adenomyosis, because the expression of CD94a (surface marker for KIR) is increased in the endometrium versus the myometrium in women with adenomyosis (17). There are a few classical NK cells CD56+CD16+ in both eutopic and ectopic endometrium in adenomyosis (20). Here, we used CD57-a terminally sulfated carbohydrate epitope (glucuronic acid 3-sulfate)-to identify NK cells. This marker identifies the final stage of NK cell maturation. Therefore, these matured CD57+ NK cells may have strong cytotoxic activity, but weak proliferative property (21). Additionally, we verified NK cells via dual immunohistochemistry staining for CD56 and CD57.

Several previous studies have demonstrated progesterone and progestin-modulated immune responses (22-26). The cyclical change of progestin in endometriums of nonpregnant women suggested hormone regulation, specifically progesterone (27). Our results showed that DNG increased the number of mature NK cells at the glandular epithelium of eutopic endometrium, which is comparable to Klinger’s findings. Oral combined contraceptive pills containing DNG have been demonstrated to increase the number of lymphocytes, monocytes and granulocytes in patients treated for one cycle (22). A similar result was seen in an animal study, where administration of DNG in rats with endometrial tissue auto-transplantation slightly increased the NK cell activity and number in peritoneal fluid cells and spleen cells (11).

Progestin treatment including megestrol acetate for endometrial hyperplasia and endometrial cancer-induced immune suppression can increase the number and activity of NK cells and other immune cells (24). NK cells are the important immune cell type in the gestational decidua. The regulation of uterine NK cells by progesterone and progestin are well-known during pregnancy (27, 28). However, the role of uterine NK cells remains unclear. Many are present during implantation, and they may be involved in the implantation mechanism (23). The potential role of uterine NK cells is that it supports the preparation of uterus for embryo implantation by producing many cytokines. Moreover, Le Bouteiller and Piccinni (27) found that uterine NK cells in early decidua could kill target cells, for example, infected maternal decidual cells, supporting the local immune responses to uterine infection (23, 27, 29). Therefore, DNG administration in patients with adenomyosis may improve the implantation process and protect the fetus from infection to pregnancies that occur after discontinuation of treatment.

Different types of progestin may affect immune responses differently (23, 30). However, there is no direct comparative study on the effect of different progestins on NK cells. This would be an interesting topic for further studies. Progesterone recruites uterine NK cells by increasing the chemokine C-X-C motif ligand (CXCL)10 and CXCL11, which has been demonstrated in an in vitro study using an endometrial organ culture system (31). In addition, hormone replacement therapy has been shown to change NK cell activity (32). In this study, DNG enhanced the NK cell number only at the glandular structure of the endometrium. There was no increase in NK cell numbers in stromal tissue of eutopic endometrium or in the ectopic endometrium. In the study conducted by Mehasseb et al. (33), they examined the expression pattern of progesterone receptor (PR)-A and PR-B and foci lesion of adenomyosis by immunohistochemistry in the endometrium of control and adenomyosis subjects. The expression of both PRs was lower in the stroma, and the inner and outer myometrium in the adenomyotic samples compared to glands. Therefore, the differential effect of DNG on various tissues may have been mediated by differential expression of PRs on adenomyotic tissue and eutopic endometrium.

DNG did not affect macrophage infiltration on eutopic and ectopic endometrium or on the myometrium from patients with adenomyosis. The effect of DNG on macrophage infiltration was different from that of GnRHa shown by Khan et al. (8). GnRHa decreased the infiltration of CD68-expressing cells in the endometrium of women with endometriosis and adenomyosis. It is possible that the local actions of DNG and GnRHa on endometriosis or adenomyosis lesions were different. The highlight of our study is that, patients were randomly assigned to two study groups by block randomization with a block size of 4. There was an equal number of participants in each study group and the cohorts had homogeneous features. This maintained the balance of the study groups and reduced selection bias. In this study we had aimed to investigate the effect of progestin on mature NK cells by immunohistochemistry, which indirectly reflect NK cell cytotoxicity. However, further studies on functional NK cells and other characteristics of NK cells, i.e. proliferation or apoptosis, are still needed.

## Conclusion

Progestin DNG administration causes an increase in uterine mature NK cells in glandular structure of eutopic endometriumn in patients with adenomyosis. The immunomodulating effect of progestin on adnenomyosis may be beneficial for implantation and fetal protection to pregnancies occurring after treatment. The enhancing effect of progestin on NK cells is differentially expressed depending on the site of immune infiltration.
